# Diagnosis-Related Outcome Following Palliative Spatially Fractionated Radiation Therapy (Lattice) of Large Tumors

**DOI:** 10.3390/cancers17172752

**Published:** 2025-08-23

**Authors:** Gabriela Studer, Tino Streller, David Jeller, Dirk Huebner, Bruno Fuchs, Christoph Glanzmann

**Affiliations:** 1 Department of Radiation Oncology, University Teaching Hospital LUKS, Spitalstrasse, 6000 Lucerne, Switzerlandchristoph.glanzmann@luks.ch (C.G.); 2 Department of Orthopedic Surgery, University Teaching Hospital LUKS, Spitalstrasse, 6000 Lucerne, Switzerland

**Keywords:** lattice radiation therapy (LRT), SFRT in large tumors, clinical results following LRT

## Abstract

Background: Lattice Radiation Therapy (LRT), a spatially fractionated stereotactic radiotherapy (SBRT) technique, has shown promising results in the palliative treatment of large tumors. The focus of our first analysis of 56 lesions ≥7 cm was on the extent of shrinkage following palliative LRT (mean 50%) and assessment of its effect duration (: mean 6 months). Herewith we present an updated analysis of our single-center LRT cohort, with a focus on LRT outcome across diagnoses and applied LRT regimens. Methods: We assessed the clinical outcome following LRT in 66 patients treated for 81 lesions between 01.2022 and 05.2025. LRT protocols included simultaneous integrated boost (sib-) LRT in 49 lesions (5 × 4–5 Gy to the entire mass with sib of 9–13 Gy to lattice vertices). Alternatively mainly in pre-irradiated and/or very large lesions—a single-fraction stereotactic LRT (SBRT-LRT) of 1 × 20 Gy to vertices only was delivered to 26 lesions. In six cases with modest response to single fraction SBRT-LRT, the sib-LRT schedule was added 4–8 weeks later. Results: The median age was 68 years (18–93). Main tumor locations were abdomino-pelvic (*n* = 34) and thoracic (*n* = 17). Histopathological diagnoses included carcinoma (*n* = 34), sarcoma (*n* = 31), and melanoma (*n* = 16). 31% of all lesions have been previously irradiated. 73% of cases underwent concurrent or peri-LRT systemic therapy. The mean/median overall survival (OS) time of the cohort was 7.6/4.6 months (0.4–40.2), 11.9/5.8 months in 16/66 alive, and 6.4/4.3 months in deceased patients, respectively. 82% of symptomatic patients reported immediate subjective improvement (PROM), with a lifelong response duration in most cases. Progressive disease (PD: >10% increase in initial volume) was found in 9%, stable disease (SD +/−10% of initial volume) in 19% of scanned lesions, and shrinkage (>10% reduction in initial volume) in 75%, with a mean/median tumor volume reduction of 51/60%. The extent of shrinkage was found to be 11–30%/31–60%/61–100% in 38/24/38% of lesions. Response rates (PD, SD, shrinkage) following the two applied LRT regimens, as well as those related to sarcoma and carcinoma diagnoses, were found to be comparable. Treatment tolerance was excellent (G0-1). Conclusions: Palliative LRT provides rapid subjective relief in ~80% of symptomatic patients. Radiologic shrinkage was stated in 75% of FU-scanned lesions, with a lifelong effect duration in most patients. LRT was found effective across histologies, with a similar extent of shrinkage in carcinoma and sarcoma following 1F SBRT- and 5F sib-LRT regimens, respectively.

## Simple Summary

Lattice Radiation Therapy (LRT), a spatially fractionated stereotactic radiotherapy (SBRT) technique, has shown promising results in the palliative treatment of large tumors. The focus of our first analysis of 56 lesions ≥7 cm was on the extent of shrinkage following palliative LRT. We herewith present an updated analysis of our single-center LRT cohort, with a focus on LRT outcome across histopathological diagnosis and applied LRT regimen. Based on the meanwhile 66 patients treated for 81 lesions, we found a failure rate to LRT in ~10% of cases, stable disease (+/−10% of pre-treatment volume) in ~10–20%, and shrinkage in ~75% of treated lesions, with a mean effect duration of > 7 months, i.e., mostly life-long in palliative patients with very large tumors with an overall survival rate of mean/median 7.7/4.6 months (0.4–40.2). In addition, the probability of shrinkage/partial remission across the most frequent histologies (carcinoma, sarcoma, and melanoma) and the extent of shrinkage in carcinomatous vs. sarcomatous lesions were similar, maybe with a lower extent of shrinkage in melanoma. The effectiveness of one fraction stereotactic LRT vs. five fraction simultaneous integrated boost-LRT was also found to be comparable.

## 1. Introduction

The palliative treatment of very large tumors remains a major challenge in oncology. These lesions are often characterized by poor vascularization, hypoxic subregions, and numerous pre-treatments, including previous radiation, limiting normal tissue tolerance of re-radiation.

Lattice Radiation Therapy (LRT), a form of spatially fractionated radiation therapy (SFRT), has emerged as a promising strategy to address these challenges. By integrating high-dose sub-volumes (“vertices”) within the gross tumor volume (GTV), LRT aims to achieve enhanced tumoricidal effects while maintaining tolerable dose distributions across critical structures. The underlying rationale is supported by radiobiological insights into immunogenic modulation, bystander effects, and vascular disruption mechanisms triggered by steep intratumoral dose gradients [1,2,3,4,5,6,7,8,9,10,11,12,13].

Since first LRT reports ~15 years ago, there is increasing but still limited clinical knowledge reported in the literature. SFRT has over decades been used for many patients with favorable and encouraging outcomes; however, this modality remains insufficiently understood. Many questions on SFRT/LRT remain open, like ideal geometric arrangements, dosage, dose-volume arrangements, and time interval for decision to repeat LRT—beside the lack of knowledge regarding underlying immuno-biological mechanisms of action. A comprehensive critical review on the recent status of clinical and preclinical studies and knowledge gaps has been recently published by Prezado et al., addressing clinical physical as well as immunobiological aspects and open questions on SFRT [1].

Our formerly published clinical outcome data demonstrated the effectiveness and favorable safety of LRT in a heterogeneous cohort of 45 patients with 56 large (≥7 cm) tumors treated in a palliative setting [2]. LRT yielded encouraging rates of symptom relief and radiologic response, persisting for a mean of 7.7 months. In the meantime, the clinical application of LRT at our center has continued to expand, supported by increasing familiarity with the LRT concept and clinical outcome and growing multidisciplinary interest. A summary of published clinical LRT results up to 2023 has been listed in Table 1 of our previous report [1]. Additional clinical reports on LRT published in the literature since then are listed in Table 1 of this work [3,4,5,6,7,8,9,10,11,12,13], mirroring an increasing interest in/spectrum of LRT indications, while orchestrated multi-center trial activities are still scant.

A recent publication informs about the formation of the Radiosurgery Society, GRID, LATTICE, Microbeam, and FLASH (GLMF) Working Groups as a framework for these efforts, focused on advancing the understanding of the biology, technical/physical parameters, trial design, and clinical practice of these new radiation therapy modalities [14].

Li et al. analyzed the effectiveness and safety of LRT in large tumors >5 cm in a systematic review and meta-analysis based on single-arm clinical studies [15]. Pooling 187 patients treated for 209 lesions out of seven eligible publications, the authors found the three-month complete response rate and partial response rate were 36.67% and 42.49%, respectively, while the three-month progressive disease rate was 7.10%. The tumor volume was reduced by 48.95%. The pooled 6-month overall survival rate was found to be 79.27%, with a median response time of 4.25 months. The pooled rates of mild and moderate-to-severe adverse events were 19.40% and 3.37%, respectively.

The following analysis represents an update of our single-center expanded patient cohort with extended FU. The focus was on consistency of LRT effectiveness across histologies and different LRT regimens applied in patients with limited or no alternative therapeutic options.

## 2. Materials and Methods

### 2.1. Patients (Table 2)

A total of 66 patients with 81 large lesions (≥7 cm diameter) were treated in palliative intent between January 2022 and May 2025. Histopathological diagnoses included carcinoma (*n* = 34), sarcoma (*n* = 31), and melanoma (*n* = 16). Median gross tumor volume (GTV) was 415 cc (range: 33–4027 cc). In 63/81 (78%) lesions, at least one follow-up (FU) imaging was available for volumetric analysis.

Prior radiation was documented in 31% of all lesions.

Systemic therapy was administered in 73% of cases; progress of lesions under systemic therapy led to referral for LRT in most cases.

Ethical approval has been applied by the Local Ethical Commission (No. AO _2023- 00061, EKNZ).

**Table 2:** Characteristics of the cohort.

Parameter	N
N patients	66
N lesions treated with LRT	81
age, mean/median (range)	65/68 y (18–93)
Localization of lesions	34 abdomino-pelvic/retroperitoneal 10 pleuro-pulmonal 7 abdomino-thoracic wall 7 sternal/pelvic bones 6 axilla 6 lower extremity 5 cervical 3 breast 2 inguina 1 upper extremity
Histopathol diagnosis of lesions	34 carcinoma 31 sarcoma 16 melanoma
Lesion size, mean/median (range)	
diameter	14/12.5 cm (7–28)
gross tumor volume (GTV)	814/415 cc (33–4027)
previous local Radiation Therapy	25/81 lesions (31%), mean/median 22/15 m (2–90) prior to LRT
Systemic Therapy	
previous +/−post	59/81 lesions
during LRT	3/81
none	19/81
LRT schedules	
(A): 1 × 20 Gy SBRT (vertices only)	N = 26
(B): 5 × 4–5 Gy/9–13 Gy SIB-LRT (to entire mass)	N = 49
(C): (A) supplemented by (B)	N = 6
LRT characteristics, mean/median (range)	
PTV2 to entire mass (0–5 mm margin to GTV)	1072/689 cc (87–4460)
PTV1 (vertices)	5.8/4.2 cc (0.35–36)
% PTV1 of PTV2	0.7/0.5 % (0.05–4)
N vertices	10/7 (1–82)
FU mean/median (range), in months	
All patients (*n* = 66)	7.7/4.6 (0.4–40.2)
Alive (*n* = 16/66)	11.9/5.8 (1.3–40.2)
Deceased (*n* = 50/66)	6.4/4.3 (0.4–36)

### 2.2. LRT

Our Lattice Radiation Treatment (LRT) protocol includes the following regimens (*):

(A) single-fraction stereotactic LRT (SBRT-LRT, *n* = 26) of 20 Gy to vertices only, as re-ported by Jiang et al. and Dincer et al. [16,17];
(B) simultaneous integrated boost LRT (sib-LRT, *n* = 49) applying 5 × 4–5 Gy to the entire mass with sib of 9–13 Gy to lattice vertices, as described by Duriseti et al. [18];
(C) combination: (A), followed by (B)—after typically 4–8 weeks: realized in only 6/81 lesions, aiming to improve treatment response to (A).

Detailed information regarding LRT contouring/planning has been reported in our former publication [2].

### 2.3. Indication for Palliative LRT/Inclusion Criteria

All patients treated with LRT were referred for palliative radiation therapy evaluation of large tumors ≥ 7 cm. In all cases surgical intervention was either not feasible or not indicated, and systemic therapy was not indicated/not possible/not effective anymore. For several of these patients with exceptionally large tumors, omission of any palliative radiotherapy has been considered upon the availability of LRT as an option at our site. Another criterion is given by the anatomical situation—i.e., the possibility to place vertices without increasing risk for normal tissue damage: flat long-axis tumors were considered not eligible and not included in the program. Also, tumors encasing neurovascular bundles or intestinal loops were strictly excluded.

(*): When we started our LRT program, regimen (A) was mainly used for very large and/or previously irradiated lesions. This single-fraction SBRT-LRT regimen was then found to be clinically similarly effective as regimen (B), while most convenient for palliative patients. This observation led us to the standard application of the single-fraction SBRT-LRT regimen (A) as the regimen of first choice—if anatomically feasible, followed by regimen (B) in cases of unsatisfactory response to regimen (A). The definition of ‘unsatisfactory’ included no or <10% of volumetric response and no subjective benefit. The condition for any further LRT (vs. best supportive care) was always the patients’ wish/interest and general condition.

### 2.4. Definition of Volumetric Response

FU-imaging was available from 63/81 lesions (78%). The following definitions of response were used (decision was against the RECIST score, which is only based on diameter of lesions):

- progressive disease (PD): >10% increase in initial volume; please note: clinically obvious PD was also counted for lesions with no FU scans;
- stable disease (SD): +/−10% of initial volume—taking the uncertainty given by edema reactions following LRT into account;
- shrinkage (>10% reduction in initial tumor volume);
- complete remission (CR): no residual tumor in diagnostic scans.

### 2.5. Follow Up

Clinical and radiological FU was conducted on an individualized basis, tailored to the individual needs of these palliative patients, i.e., FU imaging was not performed solely for analytical purposes, resulting in incomplete radiographic FU (63/81 lesions were examined with at least 1 magnetic resonance imaging or computed tomography scan, accounting for 78% of cases). In consequence, time-related volumetric change analysis was not assessable in defined time intervals. Clinical FU was assessed at our department (physical or phone call visits) and based on chart notes from other involved disciplines.

### 2.6. PROMS (Patient Reported Outcome Measure)

PROMS were collected from all patients able to state their experienced symptom changes, using a Visual Analogue Scale (VAS, ranging from 0 = no symptoms, to 10 = unbearable symptoms), Table 3.

## 3. Results

The mean/median overall survival (OS) of the cohort was 7.7/4.6 months (0.4–40.2); the respective OS of 16 alive patients (07.2025) was 11.9/5.8 (1.3–40.2), and 6.4/4.3 months for 50 deceased patients. Table 4 shows detailed characteristics of treated lesions related to histology. Sarcomatous lesions were characterized by the largest mean/median volumes.

All included lesions measured ≥ 7 cm in diameter.

### 3.1. Subjective Benefit/PROMS

Eighty-five percent of symptomatic patients (45/55) reported fast relief of symptoms, Table 3, with a life-long duration of this effect in most cases, which is supported by the objective duration of volumetric shrinkage as shown in Figure 1: 9/37 initially shrunk lesions with >1 FU scan showed re-growth; four of these nine lesions remained smaller than initially, i.e., in only 5/37 (14%) a ‘clinically relevant’ failure during the observed FU time or lifetime was found.

The FU time, based on last available imaging of depicted 37 lesions with at least 2 FU scans was mean/median 9.2/5.0 months (1–40).

### 3.2. Radiologic/Volumetric Response, Table 5, and Figure 2

Nine percent of all 81 lesions and six percent of all FU-scanned lesions, respectively, failed to respond to LRT (progressive disease, PD: increase of >10% of pre-therapeutic volume). Nineteen percent of all lesions with at least one FU scan showed stable disease (SD, defined as +/−10% volume change compared to the pre-LRT volume); the remaining 75% of FU-scanned lesions showed ≥10% shrinkage as compared to the initial volume—mostly already in the first FU (i.e., mean 2.8 months post-LRT), with a mean/median maximum tumor volume reduction of 47%/63% after 5.5 months. Regarding the extent of shrinkage, about one-third of cases reached 11–33%, 34–66%, and 67–100% shrinkage (Figure 3). The duration of volumetric response was mean/median 9.2/5.0 months (1–40) at the time of this analysis. While the number of shrunk lesions/response rate was similar between all three assessed histologies (Figure 4), the EXTENT of shrinkage seemed lower in melanomatous compared to carcinomatous and sarcomatous lesions (Figure 5)—however, this observation is to be taken with caution considering the small and unbalanced sample sizes.

**Table 5:** Volumetric response, related to histopathologic diagnosis.

Parameter	Carcinoma	Sarcoma	Melanoma	All Analyzed Lesions
**FU imaging available**	**23/34 (68%)**	**28/31 (90%)**	**12/16 (75%)**	**63/81 (78%)**
**Volumetric response to LRT**				
PD (>10% of initial cc)	3/34 (9%)	3/31 (10%)	1/16 (6%)	7/81 (9%)
SD (+/−10% of inital cc)	5/23 (22%)	5/28 (18%)	2/12 (17%)	12/63 (19%)
Shrinkage (<10% of initial cc)	17/23 (74%)	20/28 (71%)	10/12 (83%)	47/63 (75%)

**Figure 2:**
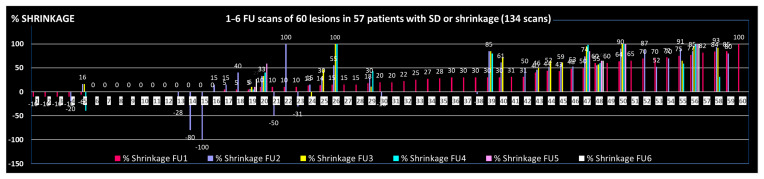
Development of the tumor volume in 60 lesions with stable disease (SD) or shrinkage, assessment based on one to six FU scans/lesion.

The volumetric response as related to the two used LRT regimens is shown in Table 6: shrinkage and extent of shrinkage (Figure 6) was found very comparable, encouraging to primarily go for the 1F SBRT-LRT regimen. Depicted in Figure 7A–D are four representative case illustrations before and after LRT using the one fraction SBRT-LRT regimen.

### 3.3. Toxicity

All patients completed the prescribed short-course LRT. Early tolerance was excellent (Grade 0–1), with only two cases of Grade 2–3 dermatitis due to tumors involving the skin; no late toxicity was assessed so far. Most patients felt some mild to moderate fatigue during a few days post LRT, several reported immediate better overall well-being.

## 4. Discussion

This update analysis of a single-center cohort treated with palliative LRT represents to our best knowledge the first report on outcome comparison of different LRT regimens and across histopathologic entities. We found similar response rates in carcinomatous, melanomatous, and sarcomatous lesions (~75% shrinkage), while the mean extent of shrinkage seemed higher in carcinoma and sarcoma (~50% of initial volume) as compared to melanoma (~28%), Figure 5; however, this is to be taken with caution considering the unbalanced and still small sample sizes. In addition, as ‘sarcoma’ is a collective term, sarcoma entity-related response to LRT is currently entirely unknown. LRT reports on melanomatous lesions are very scant and limited to case reports, not allowing any comparative analysis with their own small subgroup.

Regarding LRT in sarcoma, besides the cohort of 26 patients with 31 lesions presented here, we found two additional cohorts (53 and 15 cases) with bulky sarcomatous tumors treated with LRT [10,12]—taken together, 94 patients were treated for 110 lesions. After a median FU of 6–10 months, the rate of SD was >60% (difficult to compare, as different definitions of SD were used), with a high percentage of subjective benefit (PROM).

The previously reported duration of subjective and volumetric beneficial effects [1] has been confirmed (mean 7.7 months), i.e., a lifelong effect can be expected in most patients of such palliative cohorts.

In addition, we compared two different LRT regimens used. While the SBRT single fraction LRT (regimen A) represents the three-dimensional (3D) version of the original GRID therapy, as also reported by Jiang et al. and Dincer et al. [16,17], the sib-LRT (B) represents an innovative version of a historic classic and still widespread palliative regimen, using homogeneously calculated 5 × 4–5 Gy RT with a high-dose integrated boost (sib) as described by Duriseti et al. [18]. The sib-LRT regimen covers the entire tumor mass with an effective, broadly used palliative dose in five fractions, while -according to new immunological findings—small isolated hot spots (vertices) enforce protection of immunologically relevant cells in the microenvironment and increase building of neoantigens around the hot spots.

Comparing the two applied regimens (A) and B), different characteristics of the two sub-groups are to consider: the one fraction regimen (A) was initially mainly used for very large lesions (mean 1118 vs. 733 cc) and/or for previously irradiated tumors (46% vs. 18%), Table 6. The very comparable response, despite these unbalanced features, encouraged us to routinely start LRT treatment with regimen (A), which is most convenient for palliative patients. The SBRT-LRT single-fraction regimen may be supplemented by regimen (B) if needed, and vice versa, or by normo-fractionated external beam RT as used by several centers.

The presented update analysis may add two new findings in the field of knowledge: similar volumetric response in carcinoma and sarcoma and similar volumetric response following the two applied regimens.

Limitations of this analysis are the small unbalanced sample sizes, preventing from reliable statistical analyses. The so-named ‘carcinoma’ and ‘sarcoma’ subgroups are collective terms encompassing different disease entities—any related subgroup response specifications are missing. One of the key limitations is the erratic, unplanned imaging time intervals, as well as the sub-cohort with no FU-imaging due to situational disease-/end-of-life-related reasons. In addition, there is no complete long-term FU of the cohort available (i.e., 16/66 patients were still alive at the time of this analysis).

**Figure 1:**
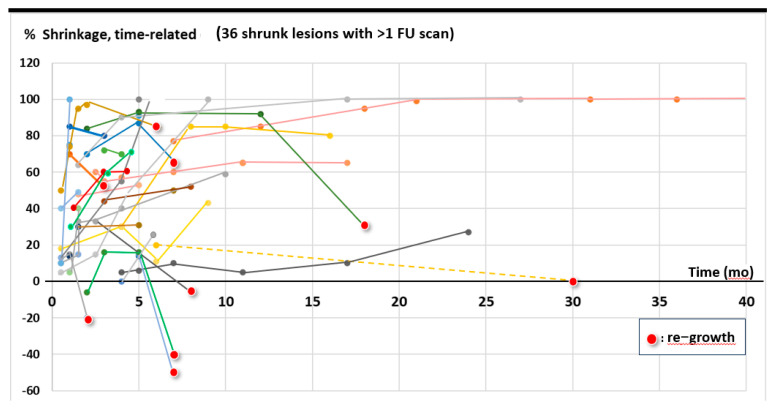
Time-related documentation of shrinkage among patients with shrunk lesions with available imaging: 9/37 initially shrunk lesions with >1 FU scan showed re-growth (red points); of importance: 4/9 lesions remained smaller than pre-LRT, i.e., only 5/37 (14%) developed ‘clinically relevant’ failure during the observed FU time or lifetime of patients.

**Figure 3:**
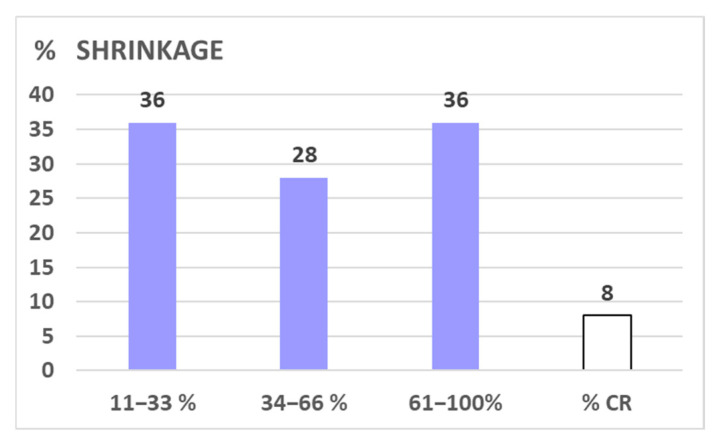
Shows the EXTENT of shrinkage in % in 47 shrunk lesions among patients with shrunk lesions and with available imaging: about one third of lesions each was found to shrink 1/3, 2/3, and ~3/3. In eight percent of our cohort, macroscopic radiologic complete remission was stated.

**Figure 4:**
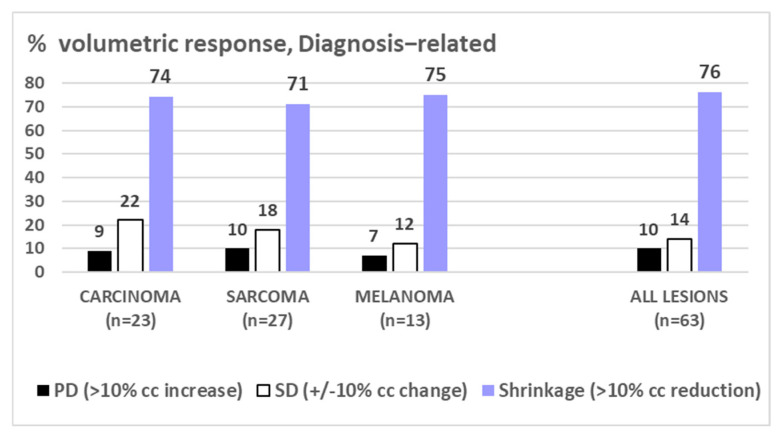
Shows very similar PERCENTAGE of response (PD/SD/Shrinkage) to LRT among the assessed histopathological diagnoses, assessment among patients with shrunk lesions or stable disease and with available imaging.

**Figure 5:**
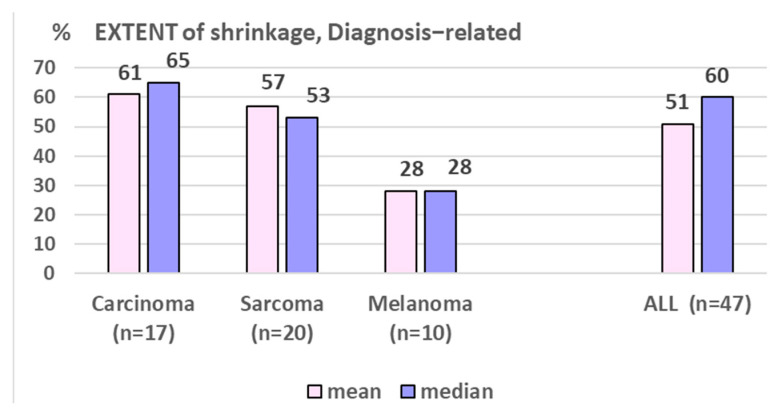
Shows the diagnosis-related EXTENT of shrinkage in %, which seems lower in melanomatous lesion. Assessment among patients with shrunk lesions and with available imaging.

**Figure 6:**
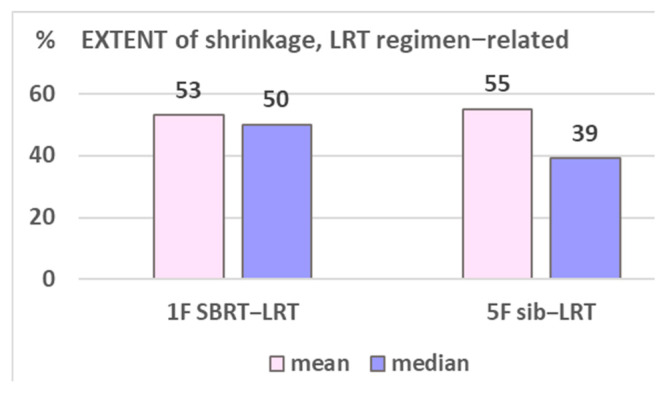
Shows the comparable LRT regimen-related EXTENT of shrinkage. Assessment among patients with shrunk lesions and with available imaging.

**Figure 7:**
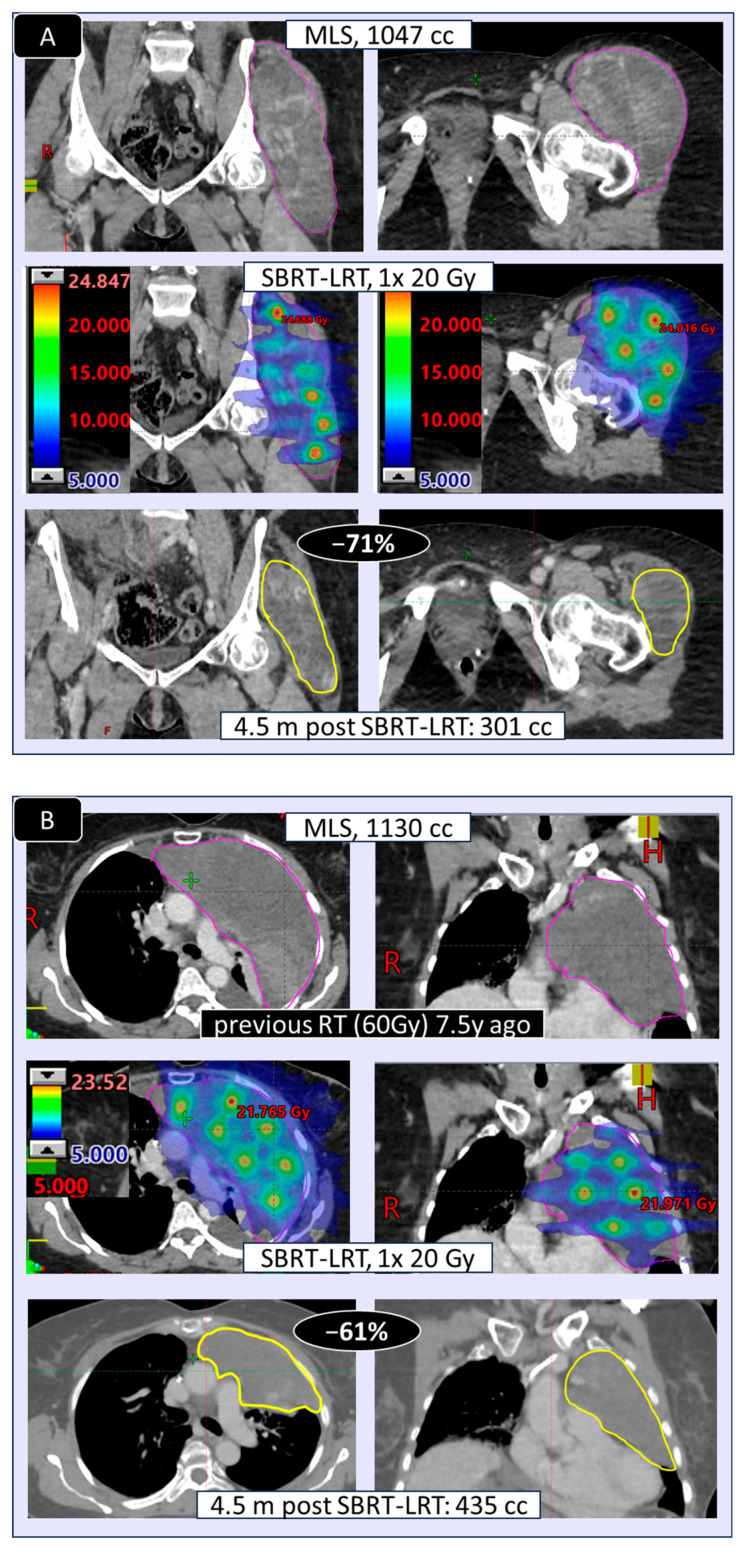

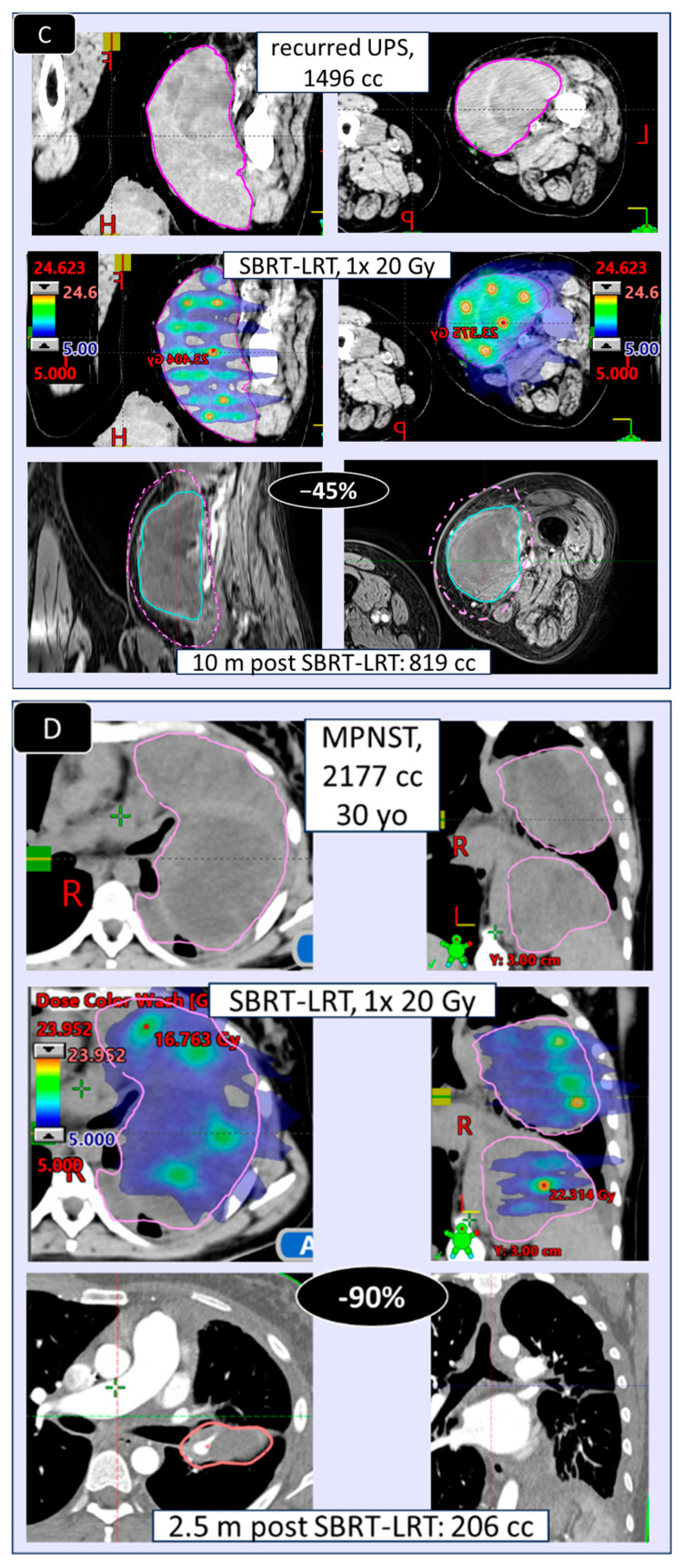
(**A**–**D**): Representative examples of large tumors treated with SBRT-LRT in one fraction (MLS: myxoid liposarcoma; UPS: undifferentiated pleomorphic sarcoma; MPNST: malignant peripheral nerve sheet tumor).

In summary, the following clinical outcome characteristics following LRT may so far hypothetically be drawn from literature and own analyses:

- ~80% of symptomatic patients experience fast subjective relief, in most cases life-long
- progressive disease/treatment failure in ~10%;
- stable disease in ~10–20% (defined as +/−10% volume change);
- shrinkage (>10% shrinkage, partial to rarely complete response) in ~>70% of cases, with the following:
  ○ mean ~50% volume reduction (extent of shrinkage);
  ○ shrinkage of 11–33%/34–66%/67–100% in ~1/3 of cases each;
  ○ complete response (CR) in ~5-<10%;
  ○ in ~15% regrowth of initially shrunk lesions to a larger than pre-treatment volume (Figure 1 and Figure 2);
  ○ response to LRT independent of pre-therapeutic size of lesions [2];
  ○ response to LRT independent of previous RT vs. RT-naïve lesions [2];
  ○ mostly fast onset of shrinkage (days to weeks) following LRT;
  ○ mean expectable shrinkage duration of 9 months, i.e., statistically a ~life-long benefit in palliative patients with large tumors (based on the assessed cohort with a mean/median OS of 7.7./4.6 mo);
  ○ similar probability of shrinkage/PR across the most frequent histologies;
  ○ similar extent of shrinkage in carcinomatous vs. sarcomatous lesions, maybe lower extent of shrinkage in melanoma—further analyses on larger samples are required;
  ○ likely similar effectiveness of 1F SBRT-LRT vs. 5F sib-LRT—further analyses on larger samples are required.

## 5. Conclusions

LRT offers a highly effective and well-tolerated palliative approach for patients with large, inoperable tumors. This extended analysis confirms prior findings of rapid symptom relief and robust tumor response in ~80% of cases with an effect duration of mean at least half a year.

In addition, LRT was found comparably effective in sarcomatous and carcinomatous lesions using a single fraction of SBRT-LRT or five fraction of sib-LRT. This is, to our best knowledge, the first clinical LRT report proving comparative response benefit across histologic subtypes and different LRT regimens.

## Figures and Tables

**Table 1:** Summary of selected clinical outcome reports over the last two years.

Autor [Ref], Y	Interval	Type	N Pat	N Lesions	Intention	Schedules	Diagnosis, Inclusion	FU	Result	≥1 FU Imaging (CT, MRI, PET-CT)
Kinj R et al. [3], 2023	-	**Case** **report**	1	1	curative boost	60 Gy, sequential 1 × 12 Gy SBRT-LRT	NSCLC	surgery	ypT0ypN0	-
Ferini G et al. [4], 2024	05.2021–11.2023	**Prosp. Cohort**	8	8	palliative	1F SBRT-LRT, sequential 5–15 F	large inoperable breast tumors	NA	4 CR, 3 PR; 100% ORR	Yes
Xu P et al. [5], 2024	06.2022–06.2023	**Retrosp. Cohort**	19	19	palliative	2–3 × 12 Gy SBRT-LRT	advanced HNC lesions > 5 cm	median 10 mo.	16/19 regression, 3 progression	Yes (1 mo. post)
Parisi S et al. [6], 2024	-	**Case** **report**	1	1	curative	1 × 10 Gy SBRT-LRT to 1 sphere, sequential 60 Gy/30 f whole breast	Inflammatory breast cancer	6 mo.	CR (plus trastuzumab)	Yes (6 mo.)
Amendola B et al. [7], 2024	01.2013–12.2021	**Retrosp. Cohort**	20	20	curative	3 × 8 Gy SBRT-LRT, sequential 21–25 × 1.8 Gy +/− boost	advanced bulky cervical cancer	median 19 mo.	+ Boost: 70% CR − Boost: 44% CR	Yes (median 19 mo.)
Liu T-F et al. F et al. [8], abstract, 2024	-	**Case** **report**	1	1	palliative	1 × 20 Gy SBRT-LRT, sequential 45 Gy/25 F	Malignant thymoma	surgery	ypT1aN×, 5 mo. FU: LC	Yes (5 mo.)
Raiden B et al. [9], abstract, 2024	06.2020–01.2024	**Retrosp. Cohort**	63	63	palliative	1 × 12–18 Gy SBRT-LRT, sequential 20 to 72 Gy/5–30 F	bulky tumors	mean 6 mo. (1–28)	55% stable disease, 34% partial response, 8% complete response, 3% progression	Yes (mean 6 mo.)
Ahmed SK et al. K et al. [10], 2024	12.2019–06.2022	**Retrosp. Cohort**	53	61	palliative	1 × 16–20 Gy SBRT-LRT, sequential consolidative EBRT	Metastatic or unresectable sarcoma	median 7.4 mo.	60% symptom relief; 35% SD 55% PR 10% PD	Yes (median 6 mo.)
Amarell K et al. [11] abstract, 2024	2019–2023	**Retrosp. Cohort**	13	13	palliative	1 × 15–20 Gy SBRT-LRT	Large tumors	median 2.8 mo.	75% SD 25% PD	8/13 (median 6.9 mo.)
Majercakova K et al. [12], 2025	2020–2024	**Retrosp. Cohort**	15	15	palliative	EBRT 45–54 Gy or 20–25 Gy/4–5F or 30 Gy/10 F, sequential 1 × 20 Gy SBRT-LRT	bulky inoperable sarcoma, no-extremity	median 10 mo.	67% stable disease (RECIST)	Yes (1–2 mo. post)
Iori F et al. [13], 2025	11.2021–08.2023	**Retrosp. Cohort**	20	20	palliative	Sib-LRT with 20/>50 Gy in 5F	solid tumors ≥4.5 cm		79% response rate @ 3 mo., 54% shrinkage	Yes (3 mo.)
own cohort, 2025	01.2022–05.2025	**Prosp. Cohort**	66	81	palliative	Sib-LRT (20–25 Gy/9–13 Gy in 5 f (*n* = 49) SBRT-LRT 1 × 20 Gy to vertices only (*n* = 26), Combination (*n* = 6)	Carcinoma (34)/Sarcoma (31)/Melanoma (16), ≥7 cm	median 6 mo. (1–40)	19% SD 75% shrinkage >10% 9% PD 8% CR	Yes (in 63/81 lesions)

**Table 3:** PROM assessment.

PROM Assessment	N Patients (N Lesions)
Asymptomatic before LRT	5 (5)
PROM not assessable	6 (6)
No change post LRT	8 (8)
Progress/worse	2 (2)
Substantial subjective benefit	45 (60)
SUMMARY	45/55 (82%) symptomatic patients able to provide PROMs experienced fast substantial durable benefit

**Table 4:** Characteristics of treated lesions.

Parameter	Carcinoma	Sarcoma	Melanoma	All
**N lesions**	**34**	**31**	**16**	**81**
metastatic	31	24	16	*n* = 71
primary	3	7	0	*n* = 10
**Initial volume (cc)**				
mean	702	922	763	780
median	343	880	248	415
range	33–3418	88–3704	66–4027	33–4027
**Initial diameter (cm)**				
mean	13	17	12	14
median	11	16	10	12.5
range	7–28	7–28	7–22	7–28

**Table 6:** Outcome, related to LRT Regimen.

Parameter (N Total)	SBRT-LRT (A) (1 F, to Vertices)	SIB-LRT (B) (4–5 F, to Entire Mass)	Combined (A) Followed by (B)
**N patients (66)**	**24**	**36**	**6**
**N lesions (81)**	**26**	**49**	**6**
**FU imaging available (64)**	**21/26 (81%)**	**39/49 (76%)**	**4/6 (66%)**
**initial volume,** mean/median cc (range)	1118/543 (33–4027)	733/435 (54–3704)	403/489 (81–1289)
**previous RT (25)**	12/26 (46%)	9/49 (18%)	4/6 (66%)
**Volumetric response to LRT**			
PD (: >10% increase in initial cc)	5/21 (24%)	1/39 (3%)	3/4 (75%)
SD (: +/−10% of inital cc)	4/21 (19%)	8/39 (21%)	0/4
Shrinkage (: >10% decrease)	12/21 (57%)	30/39 (77%)	1/4 (25%)
**EXTENT** of shrinkage, % (mean/median (range)	53/50% (15–100)	55/39% (14–100)	-
**DOD**, *n* = 49/66 (74%)	15/24 (63%)	28/36 (78%)	6/6 (100%)
**OS**, mean/median (range), in mo.	4.7/3.2 (1–23)	8.5/5.8 (0.4–40.2)	7.4/7.5 (3.7–11.6)

## Data Availability

Data may be requested – please address the corresponding author (no publicly archived dataset available).

## Abbreviations

The following abbreviations are used in this manuscript:

SFRT	Spatially Fractionated Radiation Therapy
LRT	Lattice Radiation Therapy
FU	Follow up
mo.	Month
SBRT-LRT	Stereotactic Body Radiation Therapy-LRT
SIB	Simultaneously Integrated Boost
SIB-LRT	Simultaneously Integrated Boost-LRT
F	Fraction (of radiation therapy)
PD	Progressive Disease
CR	Complete Response
SD	Stable Disease
OS	Overall survival
ORR	Overall response rate
DOD	Dead of Disease
MLS	Myxoid liposarcoma
UPS	Undifferentiated pleomorphic sarcoma
MPNST	Malignant peripheral nerve sheet tumor
